# Covert Performance for Integrated Satellite Multiple Terrestrial Relay Networks with Partial Relay Selection

**DOI:** 10.3390/s22155524

**Published:** 2022-07-25

**Authors:** Zeke Wu, Rui Liu, Haifeng Shuai, Shibing Zhu, Changqing Li

**Affiliations:** School of Space Information, Space Engineering University, Beijing 101416, China; wuzeke2022@163.com (Z.W.); shuaihf99@163.com (H.S.); sbz_zhu@sohu.com (S.Z.); lcqqcl5577@sohu.com (C.L.)

**Keywords:** integrated satellite multiple terrestrial relay network, covert communication, error detection probability, average covert communication rate

## Abstract

Integrated satellite multiple terrestrial relay network (ISMTRN) is a new network architecture that combines satellite communication with terrestrial communication. It both utilizes the advantages of the two systems and overcomes their shortcomings. However, security issues inevitably arise in the ISMTRN resulting from the broad coverage of the satellite beams and the openness of wireless communication. One of the promising methods to achieve secure transmission is covert communication technology, which has been a hot discussion topic in recent years. In this paper, we investigate the performance of covert communication in the ISMTRN with partial relay selection. Particularly, when the satellite transmits its signal to the user, we consider the scenario that the selected relay opportunistically sends covert information to the destination. Furthermore, the closed-form error detection probability and average covert communication rate are derived. Finally, numerical simulation results are provided to reveal the impact of critical parameters on system covert performance.

## 1. Introduction

Satellite communication (SatCom) is increasingly seen as an indispensable part of the next-generation wireless communications networks, which can provide a higher rate, broader coverage, lower energy consumption, more robust performance, and higher spectrum utilization [1,2]. Moreover, to make up for its shortcomings and further enhance the quality of service (QoS), SatCom is commonly combined with terrestrial communication. However, security issues arise due to the broad coverage of the satellite beams and the openness of wireless communication. In this paper, we adopt covert communication technology to solve them.

### 1.1. Related Work

As a potential future communication network architecture, the integrated satellite multiple terrestrial relay network (ISMTRN) absorbs and combines the advantages of SatCom and terrestrial communication, which guarantees the communication demands of the users and diversifies the communication ways [3,4,5]. It is becoming the research hotspot and has important practical significance for future communication development. In [6], the paper studied the performance of non-orthogonal multiple access (NOMA)-based overlay cognitive ISMTRN with secondary network selection. A hybrid satellite-terrestrial relay network (HSTRN) consisting of a satellite and multiple terrestrial nodes was considered in [7]. Moreover, the authors of [8] investigated the secure transmission in an HSTRN. In [9], the authors studied the impacts of joint relay selection and user scheduling schemes on the physical layer security for the HSTRN. The performance of an ISMTRN with the threshold-based decode-and-forward (DF) protocol was investigated in [10]. The authors of [11] established the multi-relay hybrid satellite-terrestrial systems (HSTSs) with artificial noise (AN) to investigate their reliability and security.

However, due to the broad area of satellite beams and the openness of wireless communication, communication security risks are inevitable, such as information eavesdropping, information tampering, malicious interruption interference, source location listening, and others. Traditionally, secure communication is usually achieved by high-level encryption and decryption technology. The authors of [12] used the features of quantum walk to construct a new substitution box method which played a significant role in block cipher techniques for 5G-IoT technologies. In [13], a forward secure public-key searchable encryption scheme was proposed. Besides, physical layer security (PLS) technology offers another promising direction for the security of communication networks by exploiting the inherent randomness of the physical layer wireless fading channel [1,2,9], and some other new security techniques are also widely adopted. In [14], the authors presented a method to obtain visible light communication PLS. An adaptive physical-layer key generation scheme was proposed in Smart Homes in [15]. The authors of [16] considered designs that mask the user’s identity during communication, resulting in anonymous communications, and in particular, examined the recent interest in physical layer anonymous solutions. Moreover, in [17], the authors provided a tutorial overview of the promising intelligent reflection communication (IRC) technologies and articulated multiple ways of enhancing the security of Internet-of-Everything (IoE) by the IRC. The authors of [18] discussed the challenges and opportunities of information security for next-generation integrated sensing and communication (ISAC) networks. The authors introduced the concept of physical layer anonymity and revealed some related conclusions in [19].

Nevertheless, none of those security techniques mentioned above can completely solve the security problems because eavesdroppers can obtain critical encrypted information by analyzing the eavesdropped traffic data. Thus, to further the safety of the ISMTRN, we utilize covert communication in this paper. The “prisoner model” proposed by Simmons in 1983 is a classic model in the field of covert communication. It assumed that in prison, two prisoners need to communicate. However, due to a malicious eavesdropper, Willie (passive or active listening), they could not communicate directly and needed to hide the information by some means. Hence, they exploited the randomness of the wireless communication channel and the uncertainty of noise to achieve covert information transmission to prevent the communication signal from being detected by Willie [20]. Namely, if the malicious eavesdropper cannot confirm the existence of the signal, it is difficult for it to carry out further illegal acts. Due to the unique advantages of covert communication, it has been applied in many real-life scenarios, such as covert military communications and the interconnection as well as intercommunication of the Internet of Things (IoT) [21] or UAV networks [22]. The authors of [23,24,25] investigated the information-theoretic limits of covert communication. In [26,27], a positive covert rate was realized. Moreover, interference and channel uncertainty of covert communication were discussed in [28]. Besides, in [29], the authors investigated the performance of covert communication in terrestrial relay networks. Moreover, the authors of [30] studied the covert performance under a scenario consisting of a source-destination pair, a passive warden, and multiple relays.

### 1.2. Motivation and Contributions

Recently, a wide range of research studies has been carried out to investigate covert communication in relay networks. In many of these works, covert communication is achieved with the help of channel uncertainty or jammer assistance. However, they are commonly based on separate terrestrial networks. Moreover, for multi-relay networks, relay selection is considered an effective technique to achieve spatial diversity gain, which is hardly involved in the related work. As far as we know, the performance analysis of covert communication in ISMTRN combined with relay selection has not been reported, which is innovative enough to investigate.

The above observations motivate us to investigate the covert performance of the ISMTRN with relay selection. Specifically, the key novelty and main contributions of this paper are summarized below:

1. We first pioneer the study of covert communication in the ISMTRN with the partial relay selection scheme, where the selected relay opportunistically transmits covert information;
2. Furthermore, considering the actual signal transmission situation, the statistical characteristics of the channels are given;
3. On this basis, the closed-form expression of error detection probability (EDP) under this covert communication network is deduced to obtain more in-depth insights. Besides, the average covert communication rate (ACR) is given to measure the covert performance of this system;
4. Finally, the numerical simulation results are given to analyze covert performance considerably. Moreover, observation results are summarized in detail.

The subsequent composition of this article is as follows. Section 2 consists of the system description, transmission model and problem formulation, while the performance of the considered system is investigated in Section 3. Moreover, numerical simulation results are given in Section 4. Finally, the whole article is discussed in Section 5.

## 2. System Model and Problem Formulation

This section first introduces the system we investigate. After that, the signal transmission process is analyzed. Finally, we formulate the problem we research based on this system model.

### 2.1. System Description

**Figure 1:**
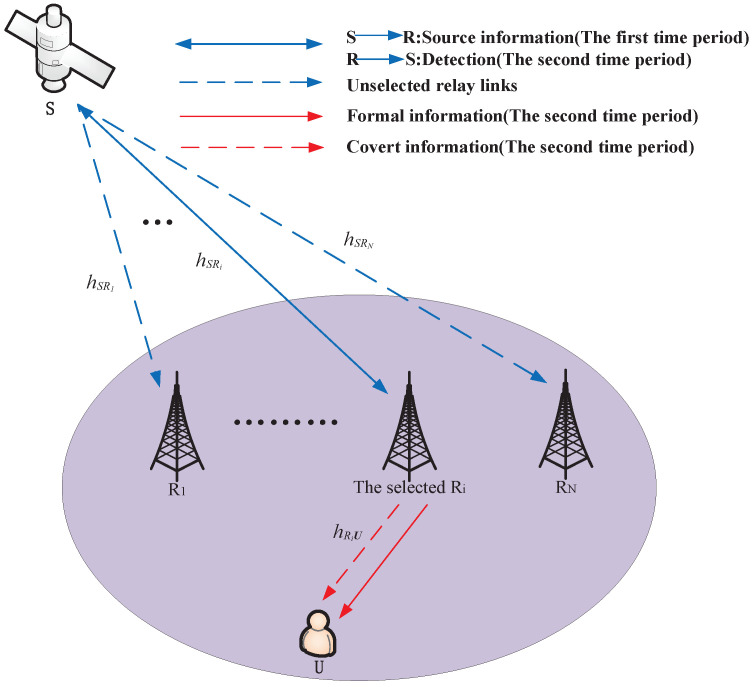
Covert communication system based ISMTRN with partial relay selection.

In Figure 1, we investigate a covert communication system based on ISMTRN with partial relay selection, consisting of a satellite *S*, a legal user *U*, and *N* DF relays, represented by $R_{i}$ (i=1,…,N). All nodes are equipped with a single antenna and work in half-duplex mode. Besides, it is assumed that the direct link between the satellite and the user cannot be achieved due to deep channel fading. In our model, we consider the scenario that while forwarding the satellite’s source information, the selected relay opportunistically transmits its information to the destination covertly. Meanwhile, the satellite acts as a monitor.

The channel coefficient of the link a→b ($a,b\in \left\{S,R_{i},U\right\}$) is defined as $h_{ab}$, which is an independent, zero-mean complex Gaussian random variable with unit variance. In order to make the analysis concrete, it is assumed that there are discrete-time channels with *T* time slots between each node, and each time slot has *n* symbol periods. Moreover, we consider that the channels within this system experience block fading, representing that channel coefficients in each time slot remain constant but vary independently from one-time slot to another. Furthermore, it is assumed that *i*th relay $R_{i}$ only knows its own $h_{SR_{i}}$ and $h_{R_{i}U}$, while *S* knows all $h_{SR_{i}}$ and *U* knows all $h_{R_{i}U}$ [29]. Finally, we assume that the satellite-terrestrial relay links are modeled by Shadowed–Rician (SR) fading, and relay-user links follow the Rayleigh fading. Generally, additive white Gaussian noise (AWGN) ($CN\left(0,\sigma^{2}\right)$) interferes with all nodes.

### 2.2. Transmission Model

In our system, a partial relay selection scheme is considered by us, which is expressed as that the relay with the highest instantaneous average signal-to-noise ratio (SNR) at *U*, denoted by $R_{m}$ (m=1,…,N), is selected as the special relay to transmit the source information from *S* [31]. Thus, for the DF relaying mentioned above, we have:

(1) $$\begin{array}{c} {\left|h_{R_{m}U}\right|}^{2}=\underset{i\in \left\{1,\ldots ,N\right\}}{\max_{︸}}{\left|h_{R_{i}U}\right|}^{2}. \end{array}$$

Therefore, the received SNR at *U* can be given by:

(2) $$\begin{array}{c} \gamma_{U}=\frac{P_{R}{\left|h_{R_{m}U}\right|}^{2}}{{\sigma_{U}}^{2}}. \end{array}$$

where $P_{R}$ is the transmit power of the selected relay, $n_{U}$ denotes the noise at *U* with $CN\left(0,{\sigma_{U}}^{2}\right)$.

Moreover, since the system realizes communication from *S* to *U* by relays, the information transfer process includes two time periods. In the first time period, *S* transmits the source information to *U* with a fixed rate, denoted by $r_{SU}$ [29]. The received signal at $R_{m}$ is given by:

(3) $$\begin{array}{c} y_{R_{m}}\left(k\right)=\sqrt{P_{ST}}h_{SR_{m}}x_{S}\left(k\right)+n_{R_{m}}\left(k\right), \end{array}$$

where $P_{ST}$ is the transmit power of *S*, k=1,2,…,n is the symbol subscript, $x_{S}$ denotes the source transmission signal of *S*, which satisfies $E\left\{{\left|x_{S}\left(k\right)\right|}^{2}\right\}=1$, and $n_{R_{m}}$ is the complex AWGN received by $R_{m}$ with $n_{R_{m}}\left(k\right)∼CN\left(0,{\sigma_{R_{m}}}^{2}\right)$.

In the second time period, whether $R_{m}$ is able to transmit information successfully is decided by two necessary conditions. One of them is that the $R_{m}$ has enough ability to decode $x_{S}$ correctly, which is expressed by:

(4) $$\begin{array}{c} \gamma_{R_{m}}=\frac{P_{ST}{\left|h_{SR_{m}}\right|}^{2}}{{\sigma_{R_{m}}}^{2}}\geq \gamma_{th}, \end{array}$$

where $\gamma_{R_{m}}$ represents the received SNR at $R_{m}$, and $\gamma_{th}=2^{2r_{SU}}-1$ is the threshold [21].

Moreover, another one is that *U* can decode the signal from $R_{m}$ successfully:

(5) $$\begin{array}{c} \gamma_{U,M}=\frac{P_{M}{\left|h_{R_{m}U}\right|}^{2}}{{\sigma_{U}}^{2}}\geq \gamma_{th}, \end{array}$$

where $\gamma_{U,M}$ is the maximum received SNR at *U*, $P_{M}$ is the maximum transmit power of $R_{m}$, and ${\sigma_{U}}^{2}$ represents the average noise power at *U*. Meanwhile, the satisfaction of the above conditions is monitored by $R_{m}$ at any time. According to Equations (4) and (5), the condition for keeping the communication link uninterrupted is obtained as:

(6) $$\begin{array}{c} \Gamma =\left\{\min\left(\gamma_{R_{m}},\gamma_{U,M}\right)\geq \gamma_{th}\right\}, \end{array}$$

when the demands set Γ is met, $R_{m}$ can transmit information successfully.

Furthermore, the two cases need to be considered, including transmitting without or with covert information. If $R_{m}$ only transmits source information to *U* without covert information, the received signal at *U* is given by:

(7) $$\begin{array}{c} y_{U_{0}}\left(k\right)=\sqrt{P_{R_{m},0}}h_{R_{m}U}x_{R}\left(k\right)+n_{U}\left(k\right), \end{array}$$

where $P_{R_{m},0}$ is the transmit power of $R_{m}$ without covert information, $x_{R}$ denotes transmission signal of source information of $R_{m}$, which satisfies $E\left\{{\left|x_{R}\left(k\right)\right|}^{2}\right\}=1$, and $n_{U}$ is the complex AWGN received by *U* with $n_{U}\left(k\right)∼CN\left(0,{\sigma_{U}}^{2}\right)$.

Since the transmission rate of *S* to *U* is fixed, $R_{m}$ must ensure $\gamma_{R_{m}}=\gamma_{th}$ at least. Therefore, the transmit power of $R_{m}$ without covert information can be expressed by:

(8) $$\begin{array}{c} P_{R_{m},0}=\frac{\gamma_{th}{\sigma_{U}}^{2}}{{\left|h_{R_{m}U}\right|}^{2}}. \end{array}$$

On the other hand, if $R_{m}$ transmits source information to *U* with covert information, the received signal at *U* is given by:

(9) $$\begin{array}{c} y_{U_{1}}\left(k\right)=\sqrt{P_{R_{m,1}}}h_{R_{m}U}x_{R}\left(k\right)+\sqrt{P_{C}}h_{R_{m}U}x_{C}\left(k\right)+n_{U}\left(k\right), \end{array}$$

where $P_{R_{m},1}$ is the transmit power of $R_{m}$ for $x_{R}$, and $P_{C}$ is the fixed transmit power of $R_{m}$ for transmitting its own covert information $x_{C}$, which also satisfies $E\left\{{\left|x_{C}\left(k\right)\right|}^{2}\right\}=1$.

When *U* receives $y_{U_{1}}$, $x_{C}$ is seen as an interference signal in the first phase of decoding $x_{R}$. Thus, the signal-to-interference-plus-noise ratio (SINR) for decoding $x_{R}$ is obtained as:

(10) $$\begin{array}{c} \gamma_{U_{1}}=\frac{P_{R_{m},1}{\left|h_{R_{m}U}\right|}^{2}}{P_{C}{\left|h_{R_{m}U}\right|}^{2}+{\sigma_{U}}^{2}}. \end{array}$$

Due to the constraints of Equation (6), $\gamma_{U_{1}}=\gamma_{th}$ should be ensured at least. Hence, the $P_{R_{m},1}$ can be denoted by:

(11) $$\begin{array}{c} P_{R_{m},1}=P_{C}\gamma_{th}+\frac{\gamma_{th}{\sigma_{U}}^{2}}{{\left|h_{R_{m}U}\right|}^{2}}. \end{array}$$

Moreover, it is apparent only when the condition $P_{R_{m},1}+P_{C}\leq P_{M}$ is satisfied, $R_{m}$ is able to transmit covert information, which is given by:

(12) $$\begin{array}{c} \Lambda =\left\{{\left|h_{R_{m}U}\right|}^{2}\geq \frac{\gamma_{th}{\sigma_{U}}^{2}}{P_{M}-\left(\gamma_{th}+1\right)P_{C}}\right\}, \end{array}$$

when the set Λ is met, the $R_{m}$ can have enough ability to realize the covert information transmission. After successfully decoding $x_{R}$, it will be stripped by *U*. Thus, the SNR for decoding $x_{C}$ is expressed as:

(13) $$\begin{array}{c} \gamma_{C}=\frac{P_{C}{\left|h_{R_{m}U}\right|}^{2}}{{\sigma_{U}}^{2}}. \end{array}$$

### 2.3. Problem Formulation

In our model, while forwarding the information of satellite *S*, the selected relay $R_{m}$ opportunistically transmits its information to the destination covertly. However, since the total communication resources are limited, the behavior of covert communication will inevitably occupy the communication resources of the primary link and be unfavorable for its communication. Therefore, the covert communication behavior at $R_{m}$ is illegal. Based on this, *S* must always detect $R_{m}$ to judge whether it transmits covert information to the user *U* or not, so that *S* can respond on time. Against this background, we perform the following analysis and present the research problem of this paper.

The received signal at *S* in the second time period can be obtained as:

(14) $$\begin{array}{c} y_{S}\left(k\right)=\left\{\begin{array}{c} \sqrt{P_{R_{m},0}}h_{R_{m}S}x_{R}\left(k\right)+n_{S}\left(k\right)H_{0}\\ \sqrt{P_{R_{m},1}}h_{R_{m}S}x_{R}\left(k\right)+\sqrt{P_{C}}h_{R_{m}S}x_{C}\left(k\right)+n_{S}\left(k\right)H_{1} \end{array}\right., \end{array}$$

where $n_{S}$ is the complex AWGN received by *S*, and $n_{S}\left(k\right)∼CN\left(0,{\sigma_{S}}^{2}\right)$. $H_{1}$ denotes $R_{m}$ transmits with covert information and $H_{0}$ is the opposite. Besides, due to the channel reciprocity, $h_{R_{m}S}=h_{SR_{m}}$. The energy detection method is adopted by *S* to probe whether $R_{m}$ is transmitting covert information, which has been proved as the best method in quasi-static fading channels in [32]. Namely, when the average power of the received signal of *S* is larger than the preset energy detection threshold, *S* considers $R_{m}$ are transmitting covert information to *U* [33]. In order to further analyze the impact of system parameters on the performance of covert communication and obtain the inspiration for optimization, we need to create a model to quantify the covert performance, namely, the detection capability of *S*. Generally, we first analyze the error detection probability of *S*.

The error detection of *S* can be divided into two categories as:

(15) $$\begin{array}{c} \begin{array}{c} P_{fa}=P\left(P_{Sr}\geq \lambda |H_{0}\right)\\ P_{md}=P\left(P_{Sr}<\lambda |H_{1}\right) \end{array}, \end{array}$$

where $P_{Sr}=1/n\sum_{k=1}^{n}{\left|y_{S}\left(k\right)\right|}^{2}$, and λ is the preset energy detection threshold. Moreover, $P_{fa}$ is the false alarm (FA) probability, and $P_{md}$ is the missed detection (MD) probability.

**Lemma** **1.** *When the demands set Γ is met, $R_{m}$ will transmit information successfully and S stars to detect. Thus, under the condition *Γ*, for a given λ, we can derive:*

(16) $$\begin{array}{c} P_{fa}=\left\{\begin{array}{c} 1,\\ FA(\lambda ),\\ 0, \end{array}\right.\begin{array}{c} \lambda <{\sigma_{S}}^{2}\\ {\sigma_{S}}^{2}\leq \lambda \leq \alpha \\ \lambda >\alpha \end{array}, \end{array}$$

(17) $$\begin{array}{c} P_{md}=\left\{\begin{array}{c} 0,\\ MD(\lambda ),\\ 1, \end{array}\right.\begin{array}{c} \lambda <\beta \\ \beta \leq \lambda \leq \gamma \\ \lambda >\gamma \end{array}, \end{array}$$

*where*

(18) $$\begin{array}{c} \alpha =P_{M}{\left|h_{R_{m}S}\right|}^{2}+{\sigma_{S}}^{2}, \end{array}$$

(19) $$\begin{array}{c} \beta =\left(\gamma_{th}+1\right)P_{C}{\left|h_{R_{m}S}\right|}^{2}+{\sigma_{S}}^{2}, \end{array}$$

(20) $$\begin{array}{c} \gamma =\left[P_{M}+\left(\gamma_{th}+1\right)P_{C}\right]{\left|h_{R_{m}S}\right|}^{2}+{\sigma_{S}}^{2}, \end{array}$$

*Moreover,*

(21) $$\begin{array}{c} FA\left(\lambda \right)=\frac{{\left[1-\exp\left(-\frac{\gamma_{th}{\sigma_{U}}^{2}{\left|h_{R_{m}S}\right|}^{2}}{\lambda -{\sigma_{S}}^{2}}\right)\right]}^{N}-{\left[1-\exp\left(-\frac{\gamma_{th}{\sigma_{U}}^{2}}{P_{M}}\right)\right]}^{N}}{1-{\left[1-\exp\left(-\frac{\gamma_{th}{\sigma_{U}}^{2}}{P_{M}}\right)\right]}^{N}}, \end{array}$$

(22) $$\begin{array}{c} MD\left(\lambda \right)=\frac{1-{\left[1-\exp\left(-\frac{\gamma_{th}{\sigma_{U}}^{2}{\left|h_{R_{m}S}\right|}^{2}}{\lambda -\left(\gamma_{th}+1\right)P_{C}{\left|h_{R_{m}S}\right|}^{2}-{\sigma_{S}}^{2}}\right)\right]}^{N}}{1-{\left[1-\exp\left(-\frac{\gamma_{th}{\sigma_{U}}^{2}}{P_{M}}\right)\right]}^{N}}. \end{array}$$

**Proof.**  See Appendix A. □

## 3. Performance Analysis

This section provides the statistical properties of the Rayleigh fading channel and SR fading channel. The exact expressions of EDP at *S* in the ISMTRN are obtained. Further, we also derive ACR to measure this covert performance of our considered system.

### 3.1. Statistical Properties of Channels

In this paper, it is assumed that all terrestrial links experience the independent identically distributed (i.i.d) Rayleigh fading. With the help of Equation (1), the probability distribution function (PDF) and cumulative distribution function (CDF) of ${\left|h_{R_{m}U}\right|}^{2}$ can be obtained as, respectively [9]:

(23) $$\begin{array}{c} f_{{\left|h_{R_{m}U}\right|}^{2}}\left(x\right)=N{\left(1-e^{-x}\right)}^{N-1}e^{-x}=N\sum_{k=0}^{N-1}\left(\begin{array}{c} N-1\\ k \end{array}\right)e^{-\left(k+1\right)x}{\left(-1\right)}^{k}, \end{array}$$

(24) $$\begin{array}{c} F_{{\left|h_{R_{m}U}\right|}^{2}}\left(x\right)={\left(1-e^{-x}\right)}^{N}=\sum_{k=0}^{N}\left(\begin{array}{c} N\\ k \end{array}\right)e^{-kx}{\left(-1\right)}^{k}. \end{array}$$

Moreover, it is inevitable to use multi-beam technology to improve the spectral efficiency in SatCom. Therefore, this factor must be considered in the channel model. For geosynchronous earth orbit (GEO) satellites, multiple beams are usually generated by array reflectors, which have a higher efficiency than direct radiation arrays. In this case, the radiation pattern of each beam is a constant, which significantly reduces the need for on-satellite processing capacity [34]. Furthermore, time division multiple access (TDMA) technology is applied to ensure that only one terrestrial user is accessed for the given time in the system.

Furthermore, the channel coefficient $h_{SR}$ between the satellite and terrestrial relay can be shown as:

(25) $$\begin{array}{c} h_{SR}=C_{SR}g_{SR}, \end{array}$$

where $g_{SR}$ represents the SR coefficient, and $C_{SR}$ denotes the radio frequency loss, which consists of free space loss and antenna gain and is expressed as:

(26) $$\begin{array}{c} C_{SR}=\frac{\lambda }{4\pi }\frac{\sqrt{G_{S}G_{R}}}{\sqrt{s^{2}+{R_{0}}^{2}}}, \end{array}$$

where λ is the carrier wavelength, *s* denotes the distance between terrestrial relay and satellite beam center, and $R_{0}\approx 35,786$ km represents the radius of geosynchronous earth orbit. Moreover, $G_{S}$ is the beam gain of the satellite, and $G_{R}$ denotes the antenna gain of terrestrial relay.

Besides, the antenna gain for terrestrial relay with parabolic antenna can be shown as [35]:

(27) $$\begin{array}{c} G_{R}\left(dB\right)≃\left\{\begin{array}{c} G_{MR},0^{\circ }<\psi <1^{\circ }\\ 32-25\log\psi ,1^{\circ }\leq \psi <48^{\circ }\\ -10,48^{\circ }<\psi \leq 180^{\circ } \end{array}\right., \end{array}$$

where $G_{MR}$ is the upper limit of the antenna gain, and ψ represents the deflection angle of the satellite. As for $G_{S}$, ϑ is defined as the angle between the terrestrial relay and the center of the satellite beam, and $\bar{\vartheta }$ represents the 3 dB angle of the satellite beam. Furthermore, the beam gain of the satellite can be given by [36]:

(28) $$\begin{array}{c} G_{S}\left(dB\right)≃G_{MS}{\left(\frac{J_{1}\left(\tau \right)}{2\tau }+36\frac{J_{3}\left(\tau \right)}{\tau^{3}}\right)}^{2}, \end{array}$$

where $G_{MS}$ represents the maximal beam gain, $\tau =2.07123\sin\vartheta /\sin\bar{\vartheta }$, $J_{1}\left(x\right)$ and $J_{3}\left(x\right)$ are the first-kind bessel function of order 1 and 3, respectively. Ideally, to obtain the best system performance, generally ϑ→0. Therefore, we can get $G_{S}=G_{MS}$, and further, $h_{SR}=C_{SR}^{Max}g_{SR}$.

From [37], the PDF of ${\left|h_{SR_{m}}\right|}^{2}={\left|C_{SR_{m}}^{Max}g_{SR_{m}}\right|}^{2}$ is given by

(29) $$\begin{array}{c} f_{{\left|h_{SR_{m}}\right|}^{2}}\left(x\right)=\alpha_{SR_{m}}{e^{-\beta_{SR_{m}}x}}_{1}F_{1}\left(m_{SR_{m}};1;\delta_{SR_{m}}x\right), \end{array}$$

where $\alpha_{SR_{m}}=\frac{1}{2b_{SR_{m}}}{\left(\frac{2b_{SR_{m}}m_{SR_{m}}}{\left(2b_{SR_{m}}m_{SR_{m}}+\Omega_{SR_{m}}\right)}\right)}^{m_{SR_{m}}}$, $\delta_{SR_{m}}=\frac{\Omega_{SR_{m}}}{\left[2b_{SR_{m}}\left(2b_{SR_{m}}m_{SR_{m}}+\Omega_{SR_{m}}\right)\right]}$, and $\beta_{SR_{m}}=\frac{1}{2b_{SR_{m}}}$. Besides, $m_{SR_{m}}$ represents the Nakagami-m parameter, $\Omega_{SR_{m}}$ denotes average power of line of sight (LOS) link and $b_{SR_{m}}$ represents half average power of the multi-path.

Generally that $m_{SR_{m}}$ is an integer, with the utilizing of [38], ${}_{1}F_{1}\left(m_{SR_{m}};1;\delta_{SR_{m}}x\right)$ is represented as:

(30) $$\begin{array}{c} {}_{1}F_{1}\left(m_{SR_{m}};1;\delta_{SR_{m}}x\right)=e^{\delta_{SR_{m}}x}\sum_{n=0}^{m_{SR_{m}}-1}\frac{{\left(-\delta_{SR_{m}}\right)}^{n}{\left(1-m_{SR_{m}}\right)}_{n}}{{\left(n!\right)}^{2}}x^{n}, \end{array}$$

where ${\left(x\right)}_{y}$ is the pochhammer function [39].

According to Equations (29) and (30), the PDF and CDF of ${\left|h_{SR_{m}}\right|}^{2}$ are obtained as:

(31) $$\begin{array}{c} f_{{\left|h_{SR_{m}}\right|}^{2}}\left(x\right)=\alpha_{SR_{m}}\sum_{n=0}^{m_{SR_{m}-1}}\frac{{\left(-\delta_{SR_{m}}\right)}^{n}{\left(1-m_{SR_{m}}\right)}_{n}}{{\left(n!\right)}^{2}}x^{n}e^{-\left(\beta_{SR_{m}}-\delta_{SR_{m}}\right)x}, \end{array}$$

(32) $$\begin{array}{c} F_{{\left|h_{SR_{m}}\right|}^{2}}\left(x\right)=1-\alpha_{SR_{m}}\sum_{n=0}^{m_{SR_{m}-1}}\sum_{k=0}^{n}\frac{{\left(-\delta_{SR_{m}}\right)}^{n}{\left(1-m_{SR_{m}}\right)}_{n}}{\left(k!\right)\left(n!\right){\left(\beta_{SR_{m}}-\delta_{SR_{m}}\right)}^{n-k+1}}x^{k}e^{-\left(\beta_{SR_{m}}-\delta_{SR_{m}}\right)x}. \end{array}$$

### 3.2. EDP

When the power constraint in the information transmission process is satisfied, namely, the set Λ is met, we set the probability of $R_{m}$ transmitting covert information as θ.

**Theorem** **1.** *For given λ and θ, the EDP can be given by*

(33) $$\begin{array}{c} \mu =P_{fa}P\left(H_{0}\right)+P_{md}P\left(H_{1}\right), \end{array}$$

*where $P\left(H_{0}\right)=1-P\left(H_{1}\right)$ and $P\left(H_{1}\right)=\theta P\left(\left.\Lambda \right|\Gamma \right)$.*

Furthermore,

(34) $$\begin{array}{c} \mu =\left\{\begin{array}{c} 1-\varepsilon \\ \left(1-\varepsilon \right)FA\left(\lambda \right)\\ \varepsilon MD\left(\lambda \right)+\left(1-\varepsilon \right)FA\left(\lambda \right)\\ \varepsilon MD\left(\lambda \right)\\ \varepsilon \end{array}\right.\begin{array}{c} \lambda <{\sigma_{S}}^{2}\\ {\sigma_{S}}^{2}\leq \lambda \leq \beta \\ \beta \leq \lambda \leq \alpha \\ \alpha \leq \lambda \leq \gamma \\ \lambda >\gamma \end{array}, \end{array}$$

where

(35) $$\begin{array}{c} \varepsilon =\theta \frac{\left[1-{\left(1-\exp\left(-\frac{\gamma_{th}{\sigma_{U}}^{2}}{P_{M}-\left(\gamma_{th}+1\right)P_{C}}\right)\right)}^{N}\right]}{\left[1-{\left(1-\exp\left(-\frac{\gamma_{th}{\sigma_{U}}^{2}}{P_{M}}\right)\right)}^{N}\right]}. \end{array}$$

**Proof.**  See Appendix B. □

Obviously, the EDP given above can be minimized by optimizing λ. From the Equation (34), according to its functional properties, it is evident that the optimal solution of λ to minimize the EDP belongs to the interval $\left[\beta ,\alpha \right]$, which has been solved by numerical simulation in Section 4.

### 3.3. ACR

Only when the conditions Γ and Λ are met at the same time, the covert rate can be achieved, which is defined as $R=\log_{2}\left(1+\gamma \right)$. With a fixed transmit power $P_{C}$, taking θ into account, the ACR of this system can be obtained as:

(36) $$\begin{array}{c} R_{AC}=\theta \int_{u}^{\infty }f_{{\left|h_{SR_{m}}\right|}^{2}}\left(x\right)dx\int_{v}^{\infty }\log_{2}\left(1+\frac{P_{C}y}{{\sigma_{U}}^{2}}\right)f_{{\left|h_{R_{m}U}\right|}^{2}}\left(y\right)dy, \end{array}$$

where $u=\frac{\gamma_{th}{\sigma_{R_{m}}}^{2}}{P_{S}}$, $v=\frac{\gamma_{th}{\sigma_{U}}^{2}}{P_{M}-\left(\gamma_{th}+1\right)P_{C}}$.

According to Equations (23) and (31) and variable *Y* substitution, Equation (36) can be derived as:

(37) $$\begin{array}{c} \begin{array}{c} R_{AC}=\theta \left[1-F_{{\left|h_{SR_{m}}\right|}^{2}}\left(u\right)\right]\frac{N}{In2}\sum_{k=0}^{N-1}\left(\begin{array}{c} N-1\\ k \end{array}\right){\left(-1\right)}^{k}e^{-\left(k+1\right)v}\\ \times \frac{{\sigma_{U}}^{2}}{P_{C}}\int_{0}^{\infty }In\left(Y+\frac{P_{C}v}{{\sigma_{U}}^{2}}+1\right)\exp\left(-\frac{\left(k+1\right){\sigma_{U}}^{2}}{P_{C}}Y\right)dY \end{array}, \end{array}$$

where $Y=\frac{P_{C}\left(y-v\right)}{{\sigma_{U}}^{2}}$.

Further, with the help of [16], the ACR can be re-written as:

(38) $$\begin{array}{c} \begin{array}{c} R_{AC}=\theta \alpha_{SR_{m}}\sum_{n=0}^{m_{SR_{m}}-1}\sum_{k=0}^{n}\frac{{\left(-\delta_{SR_{m}}\right)}^{n}{\left(1-m_{SR_{m}}\right)}_{n}}{\left(k!\right)\left(n!\right){\left(\beta_{SR_{m}}-\delta_{SR_{m}}\right)}^{n-k+1}}u^{k}e^{-\left(\beta_{SR_{m}}-\delta_{SR_{m}}\right)u}\\ \times \frac{N}{In2}\sum_{k=0}^{N-1}\left(\begin{array}{c} N-1\\ k \end{array}\right)\frac{{\left(-1\right)}^{k}\exp\left(-(k+1)v\right)}{k+1}\left[In\left(1+\frac{P_{C}v}{{\sigma_{U}}^{2}}\right)-\exp\left(\omega \right)Ei\left(-\omega \right)\right] \end{array}.\; \end{array}$$

where $\omega =\left(k+1\right)\left(v+\frac{{\sigma^{2}}_{U}}{P_{C}}\right)$ and $Ei\left(x\right)=\int_{-\infty }^{x}\frac{e^{t}}{t}dt$ is the exponential integral function.

## 4. Numerical Results

In this section, the numerical simulation results are given to investigate and analyze the impact of system parameters. Moreover, the SR fading channels parameters are shown in Table 1 [37]. The parameters of the considered system are given in Table 2 [40].

**Figure 2:**
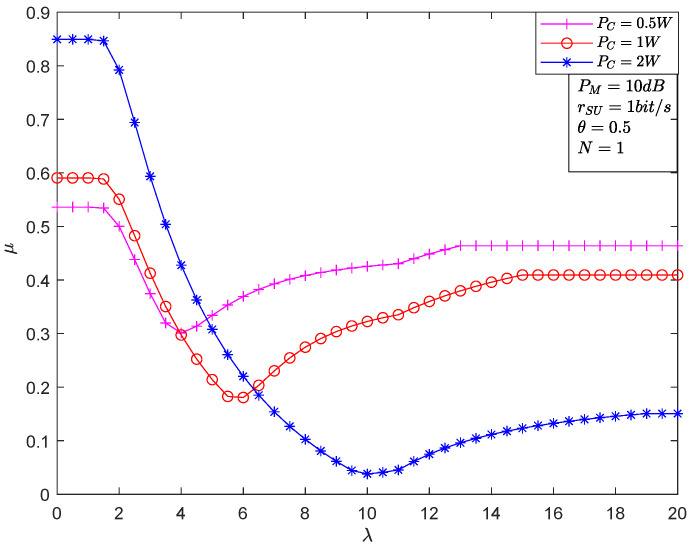
The EDP versus λ for the different $P_{C}$.

Figure 2 depicts μ versus λ for different $P_{C}$. It is obvious that the μ can be minimized by optimizing λ. Combined with Equations (21) and (22), it is easy to know that $\left(1-\varepsilon \right)FA\left(\lambda \right)$ is a minus function of λ, and $\varepsilon MD\left(\lambda \right)$ is an incremental function with respect to λ. Moreover, μ is a continuous function of λ. Therefore, the optimal solution of λ named $\lambda_{opt}$ belongs to $\left[\beta ,\alpha \right]$ which has been verified by the simulation results in this figure. Finally, we can have $\mu_{opt}=\varepsilon MD\left(\lambda_{opt}\right)+\left(1-\varepsilon \right)FA\left(\lambda_{opt}\right)$.

**Figure 3:**
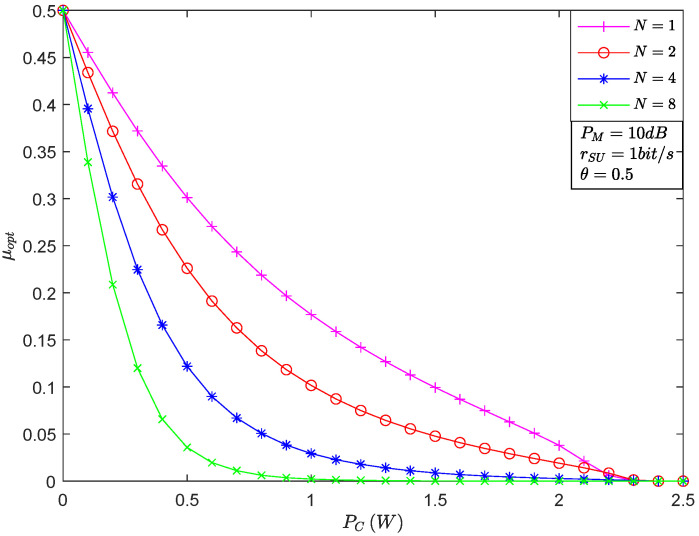
The optimal EDP versus $P_{C}$ for the different number of relays.

Figure 3 shows $\mu_{opt}$ versus $P_{C}$ for the different number of relays. In [21], the authors gave the covert constraint, namely, $\mu_{opt}\geq \min\left\{\varepsilon ,1-\varepsilon \right\}-\bar{o}$, where $\bar{o}\geq 0$. Therefore, the maximum value of $\mu_{opt}$ is ρ, which equals 0.5. Moreover, it is obvious that as the power used for covert communication $P_{C}$ gradually increases, the value of $\mu_{opt}$ decreases. It is because the enhanced power radiation used for covert communication is helpful for the detection of the satellite. All in all, the value of $P_{C}$ is determined by the covert constraint. Finally, we can also see that the increased number of relays leads to a decrease in $\mu_{opt}$, which indicates that the gain generated by the relay selection is beneficial in eliminating the uncertainty of detection at *S*.

**Figure 4:**
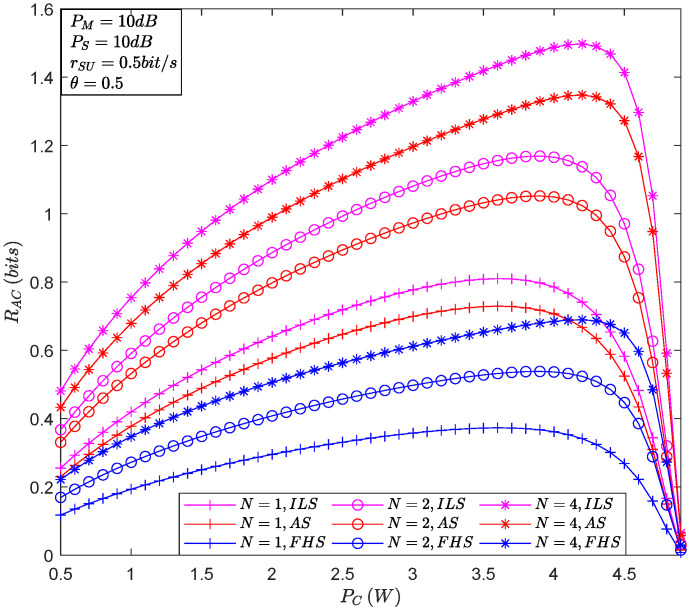
The ACR versus $P_{C}$ for the different number of relays.

Figure 4 plots ACR versus $P_{C}$ for the different number of relays. It is shown that there exists a value of $P_{C}$ that maximizes ACR, namely, ACR can be optimized by adjusting $P_{C}$. Furthermore, it is evident that as the fading of the satellite-terrestrial relay links worsens, it inevitably leads to a decrease in ACR because the degree of fading determines the proportion of on and off time for the overall system communication link. Finally, we can also see that an increase in the number of relays contributes to the rise in ACR, implying a diversity gain in relay selection.

**Figure 5:**
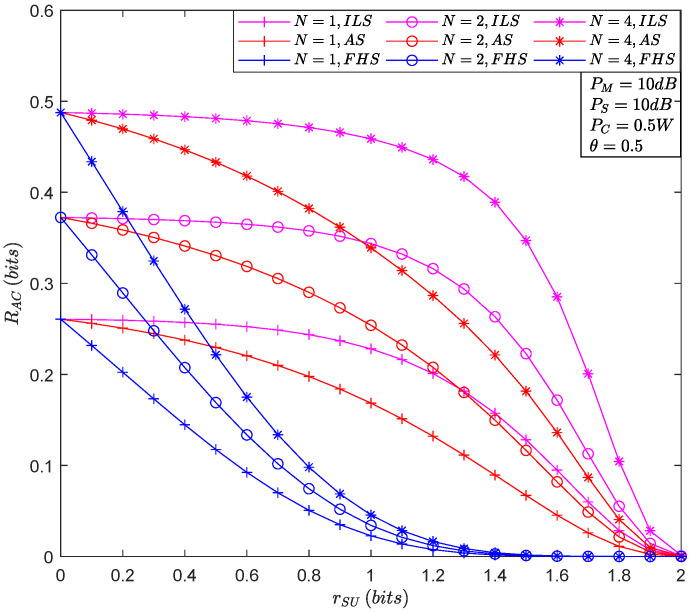
The ACR versus $r_{SU}$ for the different number of relays.

Figure 5 depicts the ACR versus $r_{SU}$ for the different number of relays. First, it is easy to see that the increase in $r_{SU}$ is detrimental to the covert communication because the enhancement in the decoding threshold increases the difficulty of keeping the communication link connected. In addition, similar to the previous conclusion, the rise in the number of relays favors the increase in the rate of covert communication, which is evident when $r_{SU}$ is small. Finally, as in the previous analysis, the ACR decreases when the satellite-terrestrial relay links’ fading deteriorates. In particular, in the ILS and AS cases, the curves become convexly decreasing, while in the FHS, the curves are concave and decreasing.

## 5. Discussion

This article investigated covert communication in the ISMTRN with relay selection. In our model, terrestrial relays sent covert information to the user. The satellite always detected terrestrial relays to prevent them from misusing communication resources to send covert information. Based on this foundation, the closed-form solutions of EDP and ACR were derived to analyze the covert communication performance. Our results showed that the gain generated by the relay selection was beneficial in eliminating the detection uncertainty. Moreover, as the fading of the satellite-terrestrial relay links worsened, it inevitably decreased ACR. Finally, a rise in the number of relays contributed to the increase of ACR, which implied the existence of the diversity gain in relay selection. 

## Figures and Tables

**Table 1:** Channel parameters.

Shadowing	$m_{j}$	$b_{j}$	$\Omega_{j}$
Frequent heavy shadowing (FHS)	1	0.063	0.0007
Average shadowing (AS)	5	0.251	0.279
Infrequent light shadowing (ILS)	10	0.158	1.29

**Table 2:** System parameters.

Parameter	Value
Satellite Orbit	GEO
Carrier Frequency	f=18 GHz
3 dB angle	$\bar{\vartheta }=0.4^{\circ }$
Maximal Beam Gain	$G_{MS}=48$ dB
Maximal Receive Antenna Gain	$G_{MR}=4$ dB

## Abbreviations

The following abbreviations are used in this manuscript:

ACR	Average covert communication rate
AN	Artificial noise
AS	Average shadow
AWGN	Addictive white Gaussian noise
CDF	Cumulative distortion function
DF	Decode-and-forward
EDP	Error detection probability
FA	False alarm
FHS	Frequent heavy shadowing
GEO	Geosynchronous earth orbit
HSTRN	Hybrid satellite-terrestrial relay network
HSTSs	Hybrid satellite-terrestrial systems
ILS	Infrequent light shadowing
IoE	Internet-of-Everything
IoT	Internet of Things
IRC	Intelligent Reflection Communication
ISAC	Integrated Sensing and Communication
ISMTRN	Integrated satellite multiple terrestrial relay network
LOS	Line-of-sight
MD	Missed detection
NOMA	Non-orthogonal multiple access
PDF	Probability distribution function
PLS	Physical Layer Security
QoS	Quality of service
SatCom	Satellite communication
SINR	Signal-to-interference-plus-noise ratio
SNR	Signal-to-noise ratio
SR	Shadowed-Rician
TDMA	Time division multiple access
UAV	Unmanned aerial vehicle

## Appendix A

Based on the Equation (15), we have known

(A1) $$P_{fa}=P\left(\left.P_{S_{r}}\geq \lambda \right|H_{0}\right).$$

Further, with the help of Equations (8) and (14), the false alarm probability can be derived as

(A2) $$P_{fa}=P\left(\left.\frac{\gamma_{th}{\sigma_{U}}^{2}}{{\left|h_{R_{m}U}\right|}^{2}}{\left|h_{R_{m}S}\right|}^{2}+{\sigma_{S}}^{2}\geq \lambda \right|\Gamma \right).$$

Then, we have known that when $\lambda <{\sigma_{s}}^{2}\;$, there is $P_{fa}=1$ and when λ>α, namely, $\gamma_{U,M}<\gamma_{th}$, there is $P_{fa}=0$. Therefore, the $P_{fa}$ can be expressed as

(A3) $$P_{fa}=\left\{\begin{array}{c} 1,\lambda <{\sigma_{S}}^{2}\\ \frac{P\left(\Gamma ,\frac{\gamma_{th}{\sigma_{U}}^{2}}{{\left|h_{R_{m}U}\right|}^{2}}{\left|h_{R_{m}S}\right|}^{2}+{\sigma_{S}}^{2}\right)}{P\left(\Gamma \right)}=\frac{P\left(\frac{\gamma_{th}{\sigma_{U}}^{2}}{P_{M}}\leq {\left|h_{R_{m}U}\right|}^{2}\leq \frac{\gamma_{th}{\sigma_{U}}^{2}{\left|h_{R_{m}U}\right|}^{2}}{\lambda -{\sigma_{S}}^{2}}\right)}{P\left({\left|h_{R_{m}U}\right|}^{2}\geq \frac{\gamma_{th}{\sigma_{U}}^{2}}{P_{M}}\right)},{\sigma_{S}}^{2}\leq \lambda \leq \alpha \\ 0,\lambda >\alpha \end{array}\right..$$

According to Equation (1) and the CDF of ${\left|h_{R_{m}U}\right|}^{2}$ as Equations (24) and (21) can be proved.

Similarly, with the help of Equations (11) and (14), the missed detection probability can be obtained as

(A4) $$P_{md}=P\left(\left.\left[\left(\gamma_{th}+1\right)P_{C}+\frac{\gamma_{th}{\sigma_{U}}^{2}}{{\left|h_{R_{m}U}\right|}^{2}}\right]{\left|h_{R_{m}S}\right|}^{2}+{\sigma_{S}}^{2}\leq \lambda \right|\Gamma \right).$$

Considering Equations (6) and (A4) can be expressed as

(A5) $$P_{md}=\left\{\begin{array}{c} 0,\lambda <\beta \\ \frac{P\left({\left|h_{R_{m}U}\right|}^{2}\geq \frac{\gamma_{th}{\sigma_{U}}^{2}{\left|h_{R_{m}S}\right|}^{2}}{\lambda -\left(\gamma_{th}+1\right)P_{C}{\left|h_{R_{m}S}\right|}^{2}-{\sigma_{S}}^{2}}\right)}{P\left({\left|h_{R_{m}U}\right|}^{2}\geq \frac{\gamma_{th}{\sigma_{U}}^{2}}{P_{M}}\right)},\beta \leq \lambda \leq \gamma \\ 1,\lambda >\gamma \end{array}\right..$$

After some steps, Equation (22) can be proved.

## Appendix B

Based on the above derivation, only when Γ is satisfied can the signal transmission link in this system remain uninterrupted. Namely, *S* can carry out its detection in the second time period, and $R_{m}$ will opportunistically transmit covert information. In the previous content, we have set the probability of $R_{m}$ transmitting covert information as θ when the set Λ is met.

From Equation (33), the EDP can be expressed as

(A6) $$\mu =P_{fa}P\left(H_{0}\right)+P_{md}P\left(H_{1}\right).$$

In Equation (A6), we have known $P_{fa}$ and $P_{md}$. Furthermore, with the help of Equations (6) and (12), the prior probability of hypothesises can be derived as

(A7) $$\left\{\begin{array}{c} P\left(H_{1}\right)=\theta P\left(\left.\Lambda \right|\Gamma \right)\\ P\left(H_{0}\right)=1-P\left(H_{1}\right) \end{array}\right.,$$

where

(A8) $$P\left(\left.\Lambda \right|\Gamma \right)=\frac{P\left(\Lambda \cap \Gamma \right)}{P\left(\Gamma \right)}=\frac{P\left({\left|h_{R_{m}U}\right|}^{2}\geq \frac{\gamma_{th}{\sigma_{U}}^{2}}{P_{M}-\left(\gamma_{th}+1\right)P_{C}}\right)}{P\left({\left|h_{R_{m}U}\right|}^{2}\geq \frac{\gamma_{th}{\sigma_{U}}^{2}}{P_{M}}\right)},$$

Substituting Equation (24) into Equations (A7) and (A8), the $P\left(H_{1}\right)$ can be obtained as

(A9) $$P\left(H_{1}\right)=\theta \frac{\left[1-{\left(1-\exp\left(-\frac{\gamma_{th}{\sigma_{U}}^{2}}{P_{M}-\left(\gamma_{th}+1\right)P_{C}}\right)\right)}^{N}\right]}{\left[1-{\left(1-\exp\left(-\frac{\gamma_{th}{\sigma_{U}}^{2}}{P_{M}}\right)\right)}^{N}\right]}\overset{\Delta }{\;=}\varepsilon .$$

Correspondingly, we can get $P\left(H_{0}\right)$ simply by Equations (A7) and (A9).

Besides, it is apparently that β<α<γ due to the power constraint of $R_{m}$. Therefore, combining Equations (16), (17), (A7) and (A9), the Equation (34) can be proved.
